# Supplementary material for: Early anteroposterior regionalisation of human neural crest is shaped by a pro-mesodermal factor
Source: eLife. 2022 Sep 26;11:e74263. doi: 10.7554/eLife.74263 (PMC9536837; doi:10.7554/eLife.74263)
Supplement: Supplementary file 10. [file elife-74263-supp10.docx]

| **Gene** | **Forward primer** | **Reverse primer** | **Roche UPL Probe** |
| --- | --- | --- | --- |
| GAPDH | 5’-agccacatcgctcagacac-3’ | 5’-gcccaatacgaccaaatcc-3’ | #60 |
| HOXA1 | 5’- gacgaccgcttcctagtgg-3’ | 5’- tcccggaagtctggtaggta-3’ | #9 |
| HOXA2 | 5’-caagaaaaccgcacttctgc-3’ | 5’-tgtgttggtgtaagcagttctca-3’ | #5 |
| HOXA3 | 5’-gacagctcatgaaacggtctg-3’ | 5’-ggtttgacacccgtgagg-3’ | #70 |
| HOXA4 | 5’-gttgccacccaagagagaac-3’ | 5’-ccaagtagtccttctcaggtatcc-3’ | #20 |
| HOXA5 | 5’-gcgcaagctgcacataag-3’ | 5’-cggttgaagtggaactcctt-3’ | #1 |
| HOXA6 | 5’-ggaaaacaagctcatcaattcc-3’ | 5’-cctgcccaggcatctactc-3’ | #26 |
| HOXB1 | 5’-ccagctagggggcttgtc-3’ | 5’-atgctgcggaggatatgg-3’ | #39 |
| HOXB3 | 5’-agctgctgaactgtccgttt-3’ | 5’-ccaggtccacgatgattttt-3’ | #3 |
| HOXB4 | 5’-aaaccaggccccttcctac-3’ | 5’-gcacacagatattcacacatacga-3’ | #45 |
| HOXB5 | 5’-aagcttcacatcagccatga-3’ | 5’-cggttgaagtggaactccttt-3’ | #1 |
| HOXB6 | 5’-tggaagctgaagaagaaactgaa-3’ | 5’-gccgggtttatgatttgttg-3’ | #12 |
| HOXB7 | 5’-ctacccctggatgcgaag-3’ | 5’-caggtagcgattgtagtgaaattct-3’ | #1 |
| HOXB8 | 5’-agctcttcccctggatgc-3’ | 5’-atagggattaaataggaactccttctc-3’ | #1 |
| HOXB9 | 5’-ccgccccttgtagaaaaat-3’ | 5’-tgtctacagtggggttgacct-3’ | #39 |
| HOXC4 | 5’-agccaattctcatccttctcc-3’ | 5’-caatgcaaaaggcctaagga-3’ | #12 |
| HOXC5 | 5’-cccgggatgtacagtcagaa-3’ | 5’-gcctgctcctctttgatctc-3’ | #25 |
| HOXC6 | 5’-tgaattcctacttcactaacccttc-3’ | 5’-atcataggcggtggaattga-3’ | #87 |
| HOXC8 | 5’-tcccagcctcatgtttcc-3’ | 5’-tgataccggctgtaagtttgc-3’ | #86 |
| HOXC9 | 5’-tcctagcgtccaggtttcc-3’ | 5’-gctacagtccggcaccaa-3’ | #70 |
| HOXC10 | 5’-aggagagggccaaagctg-3’ | 5’-agccaatttcctgtggtgtt-3’ | #19 |
| HOXD1 | 5’-caccctggtgctttccag-3’ | 5’-agagacggacttggggtagg-3’ | #57 |
| HOXD3 | 5’-tcaagaaaacacacacatacataattg-3’ | 5’-tgctgaatcctgagagagctg-3’ | #1 |
| HOXD8 | 5’-cccttgtaatcgcctgaaat-3’ | 5’-ctactgaaaataacggaacacagc-3’ | #83 |
| SOX9 | 5’-gtacccgcacttgcacaac-3’ | 5’-tctcgctctcgttcagaag-3’ | #61 |
| SOX10 | 5’-ggctcccccatgtcagat-3’ | 5’-ctgtcttcggggtggttg-3’ | #21 |
| PAX3 | 5’-aggaggccgacttggaga-3’ | 5’-cttcatctgattggggtgct-3’ | #13 |
| PAX6 | 5’- gcacacacacattaacacacttg-3’ | 5’-ggtgtgtgagagcaattctcag-3’ | #9 |
| CDX2 | 5’-atcaccatccggaggaaag-3’ | 5’-tgcggttctgaaaccagatt-3’ | #34 |
| OTX2 | 5’-ccatctccccactgtcagat-3’ | 5’-ggtcatgggataggacctctg-3’ | #4 |
| ETS1 | 5’-gcagaatgagctactttgtgga-3’ | 5’-ttgctaggtccttgcctca-3’ | #3 |
| TCF1 | 5’-cagagactcttcccggacaa-3’ | 5’-agcagattgaaggcggagta-3’ | #65 |
| LEF1 | 5’-cgacacttccatgtccaggt-3’ | 5’-atgagggatgccagttgtgt-3’ | #79 |
| SOX1 | 5’-gaagcccagatggaaatacg-3’ | 5’-ggacaaggaagggtgttgag-3’ | #66 |
| SOX2 | 5’- ttgctgcctctttaagactagga-3’ | 5’- taagcctggggctcaaact-3’ | #35 |
| SOX2 | 5’-atgggttcggtggtcaagt-3’ | 5’-ggaggaagaggtaaccacagg-3’ | #19 |
| AXIN2 | 5’-gatatccagtgatgcgctga-3’ | 5’-actgcccacacgataaggag-3’ | #56 |
| SPRY4 | 5’-ccccggcttcaggattta-3’ | 5’-ctgcaaaccgctcaatacag-3’ | #17 |
| TBXT | 5’-aggtacccaaccctgagga-3’ | 5’-gcaggtgagttgtcagaataggt-3’ | #23 |
| NKX1-2 | 5’-gtcgaagcggggaaagat-3’ | 5’-gatcctccgcatcctcct-3’ | #78 |
